# Testing Fluorescence Lifetime Standards using Two-Photon Excitation and Time-Domain Instrumentation: Fluorescein, Quinine Sulfate and Green Fluorescent Protein

**DOI:** 10.1007/s10895-018-2270-z

**Published:** 2018-07-26

**Authors:** Arne S. Kristoffersen, Svein R. Erga, Børge Hamre, Øyvind Frette

**Affiliations:** 1 Department of Physics and Technology, University of Bergen, P.O. Box 7803, Bergen, N-5020 Norway; 2 Department of Biological Sciences, University of Bergen, P.O. Box 7803, Bergen, N-5020 Norway

**Keywords:** Fluorescence, Lifetime, Fluorescein, Quinine sulfate, Green fluorescent protein, Two-photon, Time-domain

## Abstract

It is essential for everyone working with experimental science to be certain that their instruments produce reliable results, and for fluorescence lifetime experiments, information about fluorescence lifetime standards is crucial. A large part of the literature on lifetime standards dates back to the 1970s and 1980s, and the use of newer and faster measuring devices may deem these results unreliable. We have tested the three commonly used fluorophores fluorescein, quinine sulfate and green fluorescent protein for their suitability to serve as lifetime standards, especially to be used with two-photon excitation measurements in the time-domain. We measured absorption and emission spectra for the fluorophores to determine optimal wavelengths to use for excitation and detector settings. Fluorescence lifetimes were measured for different concentrations, ranging from 10^− 3^ − 10^− 5^ M, as well as for various solvents. Fluorescein was soluble in both ethanol, methanol and sulfuric acid, while quinine sulfate was only soluble in sulfuric acid. Green fluorescent protein was prepared in a commercial Tris-HCl, EDTA solution, and all three fluorophores produced stable lifetime results with low uncertainties. No siginificant variation with concentration was measured for any of the fluorophores, and all showed single-exponential decays. All lifetime measurements were carried out using two-photon excitation and lifetime data was obtained in the time-domain using time-correlated single-photon counting.

## Introduction

When a fluorophore has absorbed the necessary energy to become excited, a number of processes, e.g. fluorescence, intersystem crossing, resonance energy transfer and internal conversion, can occur - each with a certain probability, characterised by the decay rate constants *k*_*r*_ (radiative) and *k*_*nr*_ (non-radiative), and the fluorescence lifetime *τ* of a fluorophore is the average time between its excitation and its return to the ground state:

1 $$ \tau=\frac{1}{k_{r}+k_{nr}}. $$

The fluorescence lifetime is also sometimes defined as the time where the fluorescence intensity of an excited fluorophore population has decreased to 1/*e* of its initial value, which is mathematically equivalent to the average time excited.

The fluorescence lifetime is an excellent tool for investigating the dynamics of excited states and molecular interactions. For instance, a change in the lifetime for two separate fluorophore states will indicate a change in one or more of the quenching parameters described by the non-radiative decay rate constant *k*_*nr*_. Fluorophores with a reliable and well established fluorescence lifetime - a fluorescence lifetime standard - are useful for anyone performing measurements within this field, whether it is for calibration purposes, testing for systematic errors, or simply to verify and compare own lifetime measurements to the standard. It is also important to have information about the types of fluorophores that are suitable for a given instrumentation setup. Especially useful is absorption and emission spectra, as well as the lifetime itself, but also it can be important to have information about which solvents and concentrations that give the most stable and reliable results. In this paper, we have selected three fluorophores and tested their suitability to serve as fluorescence lifetime standards. In our experiments, fluorescence lifetime data were obtained using time-correlated-single-photon-counting (TCSPC). We used a laser producing pulses in the femtosecond regime, tuneable in the wavelength range 690-1040 nm, for two-photon excitation, and a detector with a range of 400-800 nm. Naturally, the fluorophores were selected to have their absorption within the range of these wavelengths, meaning that for two-photon excitation the absorption should be roughly in the range of 345-520 nm. Other selection criteria were that the fluorophores should exhibit a single-exponential decay, be readily commercially available and also have a fluorescence lifetime between 0.1-10 ns. The upper boundary for the lifetime is both to avoid a shortening from oxygen quenching, but also to avoid a pile-up effect since the repetition rate of the laser was 80 MHz, or 12.5 ns between each pulse. In addition to these criteria, the selection of fluorescein, quinine sulfate and green fluorescent protein (GFP) were also based on a quantitative literature search comprising the past five years. The present three fluorophores were amongst those with the highest number of hits in publications dealing with fluorescence and fluorescence lifetime research.

The goal of this paper is mainly to provide useful information about the present selection of fluorophores in terms of their suitability as fluorescence lifetime standards, and also gaining additional information about their properties with respect to existing literature, especially concerning two-photon excitation and lifetimes acquired in the time-domain. This instrumentation has become increasingly popular the last 10-15 years, especially in regards to working with living or fragile samples. The present study is also building on previous work with the same setup [21].

The first records of fluorescence lifetime measurements were made in the 1960s and 1970s [6, 7], however lifetime measurements from this time period were usually performed using pulse sampling oscilloscope techniques, which are not considered accurate by today’s standards. Since the late 1970s, with the introduction of high repetition rate lasers, several contributions have been made in determining lifetime standards [14, 20, 23, 33, 36]. However, many of the proposed standards have too long lifetimes to benefit today’s pico- and femtosecond instrumentation, and several were later found unsuitable due to double-exponential decays. Also, no systematic approach was done to evaluate the reliability of these lifetimes until 2007 [9], when comparisons of 13 different fluorophores were systematically examined by 9 separate laboratories in a very thorough and comprehensive work, although fluorescence was not induced by two-photon excitation in any of these measurements.

Fluorescein is available in a number of derivatives, and is widely used particularly because of a strong fluorescence signal, suitable absorption and emission wavelengths, as well as a number of other photophysical properties.

In 2014, a broad and extensive work on 20 fluorescein derivatives, which measured fluorescence lifetimes, quantum yields and emission maxima in ethanol and phosphate-buffered saline (PBS) was published [41]. There have also been several papers published in recent years, reporting on the basic fluorescent properties of various fluorescein dyes [8, 25, 27, 32, 40].

Quinine sulfate has been used as a fluorescence standard for more than 50 years [11, 13] and the photophysical properties of various quinine sulfate derivatives have also been extensively studied in the past [4, 17, 18, 26, 29]. However, fluorescence lifetime measurements are scarce after 1990.

In the 1990s, GFP from the jellyfish *Aequorea victoria* became one of the most widely studied and exploited proteins in biochemistry and cell biology [37], especially due to its unprecedented ability to serve as a marker of gene expression and protein targeting in intact cells and organisms [10, 31]. Fluorescence lifetime measurements have also been carried out [1, 30, 34, 35], however, both instrumentation and other measurement parameters differ quite significantly, and thereby also the reported fluorescence lifetimes.

A common denominator for all three fluorophores is that they are extensively used in a vast array of scientific areas, and that recent fluorescence lifetime measurements, especially using two-photon excitation and time-domain instrumentation is inadequately available in literature.

## Materials and Methods

**Fig. 1:**
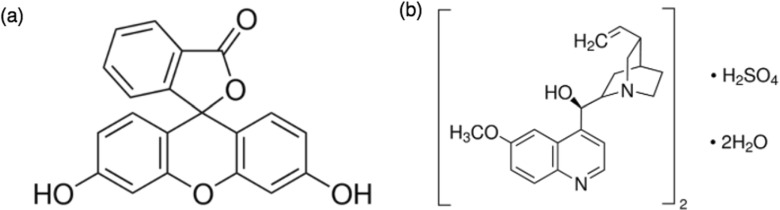
The structural formulas of fluorescein (**a**) and quinine sulfate (**b**) (from Sigma Aldrich product catalogue)

All three fluorophores were commercially available and purchased from Sigma Aldrich. Fluorescein for fluorescence, free acid (46955 SIGMA), Quinine sulfate, United States Pharmacopeia (USP) reference standard (1597005 USP), and GFP from jellyfish *Aequorea victoria*, recombinant expressed in *E. coli* (11814524001 ROCHE). The structural formulas of fluorescein and quinine sulfate are shown in Fig. 1. GFP is composed of 238 amino acid residues, and its structural formula is not shown here (see [37] for more information about the structure and composition of GFP). Ethanol and methanol of spectrophotometric grade, as well as 1 M sulfuric acid (*H*_2_S*O*_4_) were used as solvents for fluorescein, while quinine sulfate was only dissolved in *H*_2_S*O*_4_. The GFP was purchased and used in a solution form of 1 mg/ml in 5 mM Tris-HCl, 5 mM EDTA, pH 8.0. Fluorescein and quinine sulfate were prepared in concentrations of 10^− 3^ M, 10^− 4^ M and 10^− 5^ M for all solvents. Samples were prepared by placing a droplet onto a microscope slide and covered by a 0.17 mm thick glass. All measurements were performed in room temperature (20^∘^C).

### Instrumentation

A Ti:Sapphire laser (Coherent Chameleon Ultra), generating femtosecond pulses (pulse width 140 fs) at an 80 MHz repetition rate (12.5 ns between each pulse) was used for two-photon excitation of the samples. The laser is tunable in the wavelength range 690-1040 nm, with an average output power of 4 W at 800 nm. An electro-optical modulator (EOM) controlled the intensity of the laser, which in turn was guided by mirrors into a confocal inverted microscope (Leica TCS SP5) and focused by a water immersion 63× objective with a numerical aperture NA = 1.2 and a working distance of 0.22 mm. The sample was scanned at a line frequency of 400 Hz and fluorescence was detected by a built-in photomultiplier tube (PMT) detector with a range of 400-800 nm. Line, frame, and pixel clock signals were generated and synchronized by another PMT detector (Hamamatsu R3310-02) and linked via a time-correlated single-photon counting (TCSPC) imaging module (SPC-830, Becker-Hickl, Berlin, Germany) to generate fluorescence lifetime raw data. Absorption spectra were measured using a spectrophotometer (Shimadzu UV-2450) and data was collected using the software UVProbe (v.2.42). Fluorescence emission spectra were measured using the lambda scan feature in the Leica LAS software.

### Absorption and Emission Measurements

All samples were diluted to a concentration of 10^− 5^ M prior to both absorption and emission spectrum measurements. Before beginning the absorption spectrum measurements, a sample of distilled water was used to determine a baseline measurement with a very low absorption. The samples were placed in a cuvette and inserted in the spectrophotometer, which scanned in 1 nm steps from 200-700 nm. The lambda scan performs a fluorescence intensity measurement step-wise through a pre-determined detector wavelength interval. Since two-photon excitation is a non-linear process [42], the absorption spectra were used to help find a suitable excitation wavelength. For fluorescein, we used an excitation wavelength of 900 nm and a detector range of 440-700 nm. For quinine sulfate, excitation was at 800 nm and detector range set to 400-600 nm. For GFP, excitation was also at 800 nm, while the detector range was set to 450-590 nm. For all emission measurements the detector bandwidth was set to 30 nm and step size to 3 nm to achieve both a strong fluorescence signal and a smooth spectral curve.

### Fluorescence Lifetime Measurements

The spatial resolution of the fluorescence lifetime measurements were 128 × 128 pixels in order to achieve a good time resolution of 256 channels. Fine-tuning of the distance between sample and objective was necessary for each measurement to find focus, easily identified using a live scan showing the fluorescence intensity in real time. Each measurement was started by first initiating a live mode in the Leica LAS software, making sure that the sample was in focus and then starting the TCSPC module measurement, with an acquisition time of at least 1 minute, or until at least 10000 photons were collected for the brightest pixel. The laser intensity was adjusted to achieve a sampling rate of around 10^6^ photons per second. A higher sampling rate increases the risk of the so-called pile-up effect [5], where more than one photon is collected between two laser pulses. To ensure statistical significance, each sample was measured three times at different locations within the sample, then a new droplet of the same fluorophore was measured in the same way two times, so that each fluorophore-solvent-concentration combination was measured nine times. Lifetime values and uncertainties were calculated as an average of all nine measurements.

### Data Analysis

**Fig. 2:**
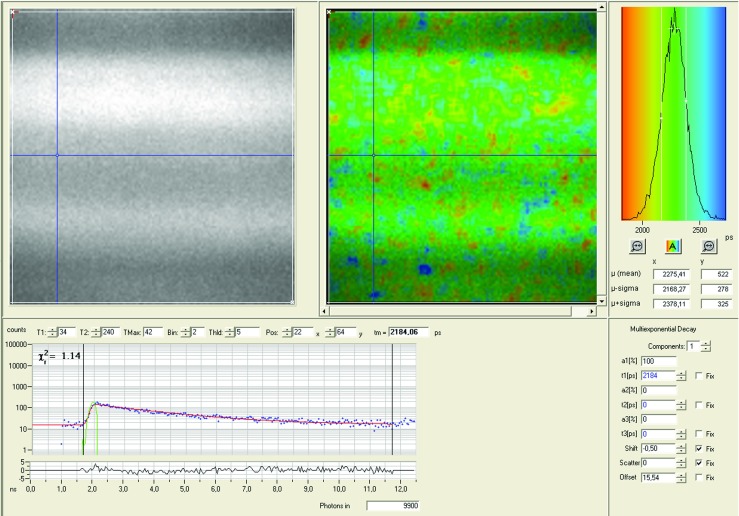
The upper left shows the fluorescence intensity for quinine sulfate dissolved in sulfuric acid at 128x128 pixels, the lifetime image next to it is color-coded from the curve in the top right, which shows the distribution of lifetimes for the whole image. The lower part of the Figure shows the exponential decay from detected photons for a single pixel in the image indicate by the blue cross-hair. The green curve is the instrument response function, and the *χ*^2^ is 1.14, indicating a very good fit for a single-exponential decay

After collecting the fluorescence lifetime data with the TCSPC module, the software SPCImage (Becker-Hickl, Berlin, Germany) was used to calculate the decay matrix. The collected photons for each pixel are stored as a histogram, and a decay curve is fitted to this data to calculate the fluorescence lifetime. A *χ*^2^ value indicates how well the fit is (1 indicates a best fit), and the matrix contains the calculated lifetimes for all the pixels in the image. Both lifetime data and the intensity (number of photons collected per pixel) was exported and further dealt with in MatLab*Ⓡ* to make plots, calculate mean lifetimes and standard deviations. Figure 2 shows the lifetime images and decay curves of quinine sulfate in sulfuric acid, from the SPCImage software.

## Results and Discussion

### Absorption and Emission

**Fig. 3:**
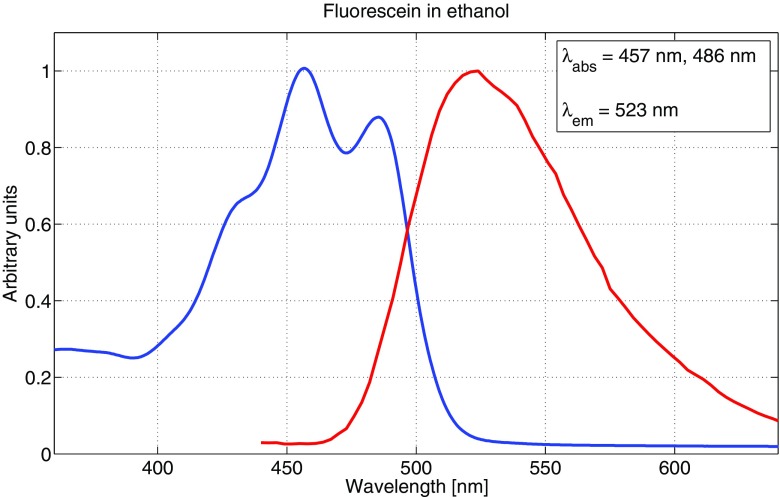
Absorption and emission spectrum for a 10^− 5^ M concentration of fluorescein in ethanol. *λ*_abs_ indicates both peaks in the absorption spectrum. The blue curve represents absorption and the red curve represents emission. Both curves are normalised to unity

**Fig. 4:**
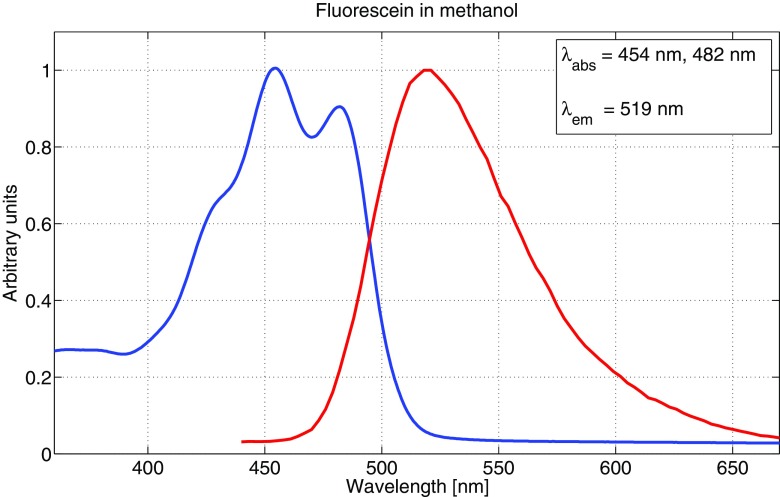
Absorption and emission spectrum for a 10^− 5^ M concentration of fluorescein in methanol. *λ*_abs_ indicates both peaks in the absorption spectrum. The blue curve represents absorption and the red curve represents emission. Both curves are normalised to unity

**Fig. 5:**
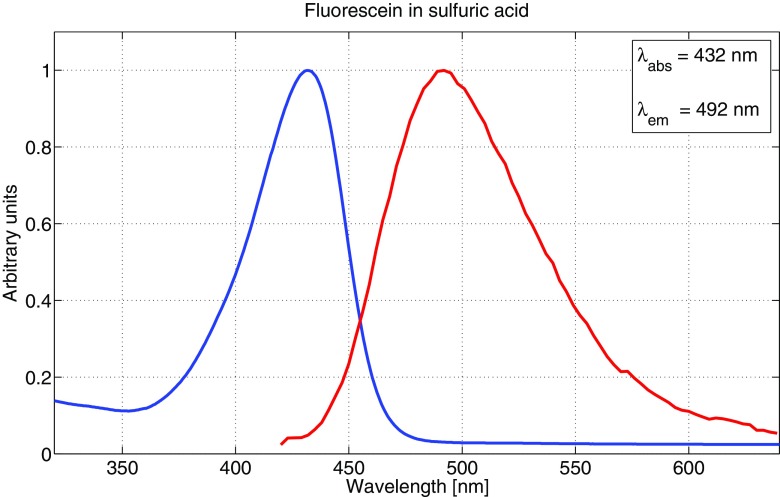
Absorption and emission spectrum for a 10^− 5^ M concentration of fluorescein in 1 M sulfuric acid. The blue curve represents absorption and the red curve represents emission. Both curves are normalised to unity

The main reason to measure absorption and emission spectra for the fluorophores was to be able to make well-founded decisions in choosing excitation wavelengths as well as setting detector intervals for fluorescence. All fluorophores were diluted to 10^− 5^ M concentrations for both absorption and emission measurements, to avoid possible aggregating of molecules as well as still having a sufficient fluorescence signal strength. It should be noted that the emission spectra were not corrected in regards to detector sensitivity variation at different wavelengths, which may cause a slight shift in emission peaks. However, this was not crucial to our measurements since the detector range was relatively broad for all samples. Figures 3, 4 and 5 show the absorption and emission spectra of fluorescein dissolved in ethanol, methanol and sulfuric acid, respectively. The spectra for ethanol and methanol are very similar for both absorption and emission, while dissolved in sulfuric acid, the spectra are shifted towards the blue end of the visible spectrum by 20-30 nm. Also, there are two absorption peaks for ethanol and methanol, compared to one for the sulfuric acid solution. Multiple absorption (or emission) peaks are in general attributed to vibronic transitions, which describes simultaneous changes in electronic and vibrational energy levels of a molecule [12, 22].

Since there is a large range of different fluorescein derivates in use [41], the absorption and emission spectra also vary substantially. Our measurements are, however, in good accordance with recently published results of free acid type fluorescein [2, 15, 19], with two characteristic absorption peaks around 450 and 480 nm, and an emission peak around 520 nm for both ethanol and methanol solutions. The present work is to our knowledge the first published on fluorescein absorption and fluorescence properties in a sulfuric acid solution. We also note that fluorescein did not completely dissolve in distilled water, and therefore we do not include any measurement results using this solvent.

**Fig. 6:**
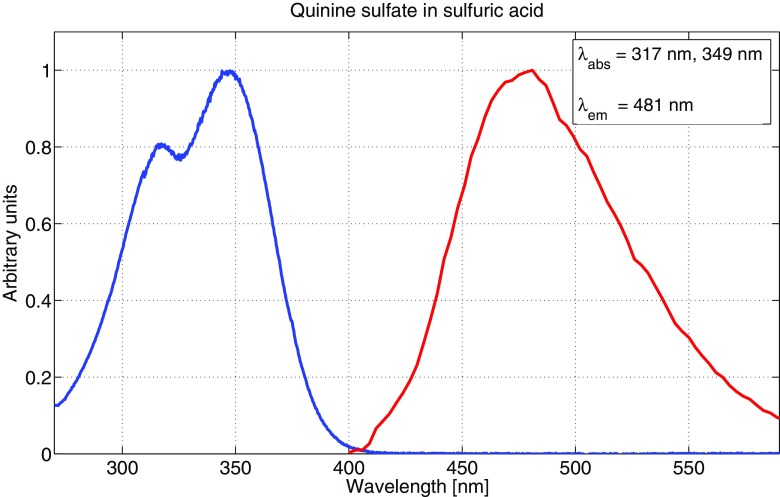
Absorption and emission spectrum for a 10^− 5^ M concentration of quinine sulfate in 1 M sulfuric acid. *λ*_abs_ indicates both peaks in the absorption spectrum. The blue curve represents absorption and the red curve represents emission. Both curves are normalised to unity

Quinine sulfate does not completely dissolve in water, ethanol or methanol, and is according to literature almost exclusively dissolved in sulfuric acid. Figure 6 shows the absorption and emission spectra for quinine sulfate dissolved in sulfuric acid. Absorption peaks at around 320 nm and 350 nm are in excellent agreement with both recent and earlier results [13, 16, 39]. We do, however, report a slightly red-shifted fluorescence emission maximum, at 481 nm, compared to typically around 450 nm found in literature [16, 39]. The emission peak in Fig. 5 is broader compared to those reported in literature, although the intensity at 450 nm is still quite high, at about 80% of the peak value.

**Fig. 7:**
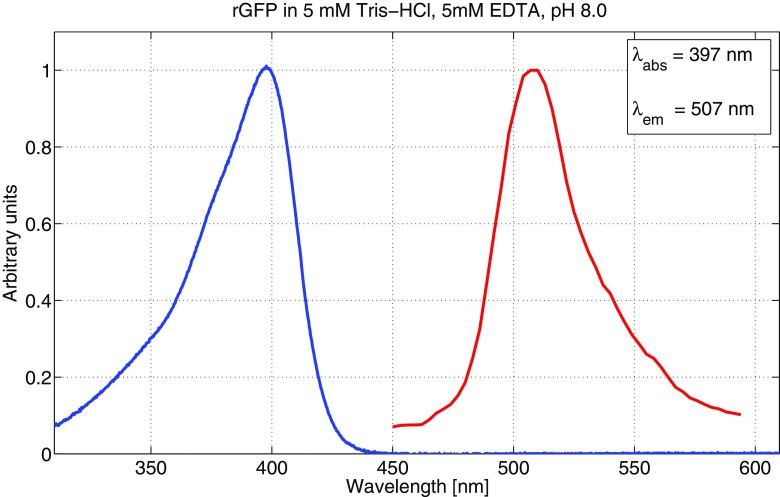
Absorption and emission spectrum for 1 mg/ml green fluorescent protein in 5 mM Tris-HCl, 5 mM EDTA, pH 8.0 solution. The blue curve represents absorption and the red curve represents emission. Both curves are normalised to unity

The absorption and emission spectra for the (GFP) is shown in Fig. 7. Both the absorption peak at 397 nm and the emission at 507 nm are in excellent agreement with previous measurements of rGFP [10], and wild type [37], who reported 395 nm and 509 nm, and 395-397 nm and 504 nm, respectively, although they also reported a minor absorption peak at 540 nm and an emission shoulder at 540 nm, which were not pronounced in our results.

### Fluorescence Lifetimes

Fluorescein fluorescence lifetimes were measured in concentrations of 10^− 3^ M, 10^− 4^ M and 10^− 5^ M in solutions of ethanol, methanol and sulfuric acid. Quinine sulfate lifetimes were measured within the same concentration range, but only dissolved in sulfuric acid as it did not dissolve satisfactory in ethanol and methanol. We also attempted to dissolve both fluorescein and quinine sulfate in distilled water, however, none dissolved properly and since the following lifetime measurements were highly variable, the results are not included here. (GFP) fluorescence lifetimes were measured only for the original 1 mg/ml (GFP) in 5 mM Tris-HCl, 5 mM EDTA, pH 8.0 solution. All lifetimes are given in nanoseconds (ns) with an uncertainty of ± one standard deviation.

Our fluorescein fluorescence lifetime measurements were performed using an excitation wavelength of 900 nm and a detector interval of 440-770 nm. The lifetimes were measured for three concentrations (10^− 3^, 10^− 4^ and 10^− 5^ M) dissolved in ethanol, methanol and sulfuric acid. As shown in Table 1, the lifetimes are remarkably similar for all solvents and concentrations, with very low uncertainties. It has been previously reported no change in lifetimes within a concentration range of 10^− 5^-10^− 7^ M in EtOH and PBS buffer [41], and our results show that there is no dependency between lifetimes and concentrations as high as 10^− 3^ M in ethanol, methanol and sulfuric acid. As fluorescein exists in many forms, earlier results also vary, however, most earlier measurements indicate lifetimes near 4 ns, such as for fluorescein dianion form dissolved in water, EtOH and MeOH [24, 25] and 3.2-3.9 ns for fluorescein in thriethanolamine-buffered saline (TBS) with no variations for concentrations up to 10^− 3^ M [3], where measurements began to lose stability likely due to homo-FRET. Our fluorescein fluorescence lifetimes varied from 3.3 ns dissolved in sulfuric acid to 3.4 ns in methanol and 3.6 ns in ethanol, with only very small variations with concentration. All measurements exhibited single-exponential decays.

**Table 1:** Fluorescein fluorescence lifetimes [ns]

	10^− 3^ M	10^− 4^ M	10^− 5^ M
Ethanol	3.62 ± 0.10	3.59 ± 0.10	3.49 ± 0.12
Methanol	3.43 ± 0.10	3.35 ± 0.10	3.38 ± 0.12
*H*_2_S*O*_4_	3.35 ± 0.09	3.34 ± 0.10	3.27 ± 0.14

The fluorescence lifetimes of quinine sulfate dissolved in sulfuric acid for 10^− 3^ M, 10^− 4^ M and 10^− 5^ M concentrations are shown in Table 2. The lifetimes increase only slightly with dilution, although the uncertainty also increases by a similar value. All three conditions showed single-exponential decays, which is remarkable because several earlier works dating 25-35 years back deemed quinine sulfate unsuitable as lifetime standards because of exhibiting double exponential decays [26, 28]. This may well be the reason that fluorescence lifetime measurements of quinine are basically absent from literature since the early 1990s.

**Table 2:** Quinine sulfate fluorescence lifetimes [ns]

	10^− 3^ M	10^− 4^ M	10^− 5^ M
*H*_2_S*O*_4_	2.26 ± 0.10	2.28 ± 0.13	2.34 ± 0.18

The (GFP) fluorescence lifetime was only measured for the original solution of 1 mg/ml (GFP) in 5 mM Tris-HCl, 5 mM EDTA with a pH of 8.0. Our measurements gave a lifetime of 2.8 ns, as shown in Table 3. This agrees very well with earlier results, with 2.8 ns for a deprotonated species [34], and also very good with 2.7 ns reported for a GFP-Rac2 [38] and 2.9 ns for a recombinant GFP [35]. The latter, however, also reported double exponential decay, with the 2.9 ns component contributing 90% of the total fluorescence. All our measurements of GFP fluorescence lifetimes produced single-exponential decays.

**Table 3:** Green fluorescent protein fluorescence lifetime [ns]

	5 mM EDTA, pH 8.0
5mMTris − HCl	2.79 ± 0.07

## Conclusions

To determine whether a fluorophore is suitable to use as a fluorescence lifetime standard, the two most important criteria is that it exhibits a single exponential decay and that it does not vary within at least two orders of magnitude in concentration. It is also important that the fluorophore has a strong fluorescence signal, and that it is completely soluble in a solvent. As a prerequisite, it should have absorption and emission peaks in the visible spectrum, as most lasers and detectors operate within this region, although this is of course dependent on what kind of fluorescence lifetime system one is using. All three tested fluorophores meet these criteria, since they were selected on this premise, although we were still able to rank them based on how well we think they are suitable as fluorescence lifetime standards. We found fluorescein to be the most suitable of the three fluorophores considered in this work, since the fluorescence lifetimes were stable with low uncertainties for all the tested solvents and concentrations. Fluorescein is also extensively used in a wide range of research fields, and is relatively affordable in large quantities. Quinine sulfate also yielded reliable lifetimes, however, its use was restricted with respect to solvent as it dissolved properly only in sulfuric acid. This makes it less suitable both for experimental work and as a lifetime standard compared to fluorescein, especially since sulfuric acid is a more hazardous chemical to work with than ethanol and methanol. (GFP) from the jellyfish *Aequorea victoria*, recombinant expressed in *E. coli*, did also produce very good fluorecence lifetime results, however, the suitability as a reference standard is not at the level of fluorescein because it is not readily available to dissolve in various solvents, and it is also more expensive compared to both fluorescein and quinine sulfate.
